# ASO Author Reflections: The FIBA Score—A Novel Scoring System to Predict Outcomes in Hepatocellular Carcinoma After Surgical Resection or Transarterial Chemoembolization

**DOI:** 10.1245/s10434-022-12959-2

**Published:** 2022-12-24

**Authors:** De-Hua Chang, Yu-Dong Xiao, Yao Tong

**Affiliations:** 1 Department of Diagnostic and Interventional Radiology, University Hospital Heidelberg, Heidelberg, Germany; 2 Department of Radiology, The Second Xiangya Hospital of Central South University, Changsha, China

## Past

After curative resection, the prognosis for hepatocellular carcinoma (HCC) varies greatly, with a 30–70% 5-year survival rate. Likewise, the same can be said for patients with unresectable HCC treated with transarterial chemoembolization (TACE). To more accurately evaluate patient prognosis, several grading systems have been created. Most scoring systems only focus on a few aspects of this complex disease, resulting in a limited prognostic value.^1,2^ As a result, scoring systems are still not frequently used in clinical practice. Therefore, our goal was to create a scoring system that was simple to use and took into account the three key prognostic factors: liver function, systemic inflammation, and tumor features.

## Present

Our study included a training cohort to create a novel prognostic scoring system (named the function-inflammation-burdenalpha-fetoprotein [FIBA] score) and two validation cohorts to test its predictive value.^3^ The FIBA score is simple to use in clinical practice because it is made up of six commonly measured parameters: albumin, total bilirubin, lymphocyte count, α-fetoprotein (AFP) level, diameter of the largest tumor, and number of tumors. It has been demonstrated to be useful in detecting HCC patients at high risk of having a poor outcome and can be used in patients who underwent curative surgical resection or TACE as the initial therapy.

In terms of prognostic predictive value, the FIBA score outperforms 16 other established scoring systems in both the training and validation cohorts.

## Future

In our Asian study cohort, the most common etiology for the development of HCC was chronic hepatitis B infection. Patients with alternative causes of HCC, such as chronic hepatitis C, alcoholic hepatitis, or non-alcoholic steatohepatitis, which is widespread in Western nations, have a different prognosis. Patients receiving systemic therapy were also excluded from our study. Here, additional validation of the FIBA score is required to support our findings and permit broader applicability.
